# Bidrectional associations between gait speed and cognitive performance in older adult women: the Study of Women's Health Across the Nation

**DOI:** 10.1002/alz70860_105707

**Published:** 2025-12-23

**Authors:** Jillian S. Baker, Lauren P. MacConnachie, Alexis Reeves, Michelle M. Hood, Carol A. Derby, Bradley M. Appelhans, Sherri‐Ann M. Burnett‐Bowie, Kelley Pettee Gabriel, Aleda M. Leis, Kelly R. Ylitalo, Carrie A. Karvonen‐Gutierrez

**Affiliations:** ^1^ University of Michigan, Ann Arbor, MI, USA; ^2^ Stanford University, Stanford, CA, USA; ^3^ Department of Neurology, and Department of Epidemiology and Population Health, Albert Einstein College of Medicine, Bronx, NY, USA; ^4^ Rush University, Chicago, IL, USA; ^5^ Harvard Medical School, Boston, MA, USA; ^6^ The University of Alabama at Birmingham, Birmingham, AL, USA; ^7^ Baylor University, Waco, TX, USA

## Abstract

**Background:**

Many women experience declines in physical function across and beyond menopause. While there is an established bidirectional association between gait speed and cognition, longitudinal studies are needed to quantify the magnitude of these relationships. Additionally, prospective studies, particularly among older adults, are prone to selection bias due to differential loss‐to‐follow‐up. We leveraged data from the Study of Women's Health Across the Nation (SWAN) to quantify the associations between gait speed and cognitive function, accounting for loss‐to‐follow‐up.

**Methods:**

SWAN is a multi‐ethnic, community‐based cohort of women followed through menopause into older adulthood. At study visits in 2012‐13 (V13) and 2015‐16 (V15), participants completed a timed four‐meter walk to assess gait speed and tests of working memory, processing speed, and verbal memory. A summary measure of cognition, the cognitive z‐score, averaged z‐scores across cognitive tests. Linear regressions were employed to predict V15 cognitive z‐score from V13 gait speed and vice versa, adjusting for demographic and socioeconomic characteristics. Inverse probability weights for missingness due to death, stroke, poor physical function, and other reasons adjusted for differential loss‐to‐follow‐up from baseline.

**Results:**

The sample included 1,258 women who completed gait speed and cognitive tests at visits 13 and 15, with a mean age of 65.5 years (SD=2.7) at V15. Half the sample was White, 23.5% Black, 12.2% Chinese, 10.8% Japanese, and 3.5% Hispanic. Adjusting for race, education, age, and financial strain, faster gait speeds at V13 were associated with higher global cognitive z‐scores at V15 (β=0.20, 95%CI:0.05,0.35) but higher global cognitive z‐scores at V13 were not significantly associated with faster gait speeds at V15 (β=0.017, 95%CI:‐0.002,0.036). Adjusting for covariates and loss‐to‐follow‐up, weighted models indicated gait speeds at V13 were positively associated with global cognitive z‐score (β=0.22, 95%CI:0.06,0.37) and global cognitive z‐scores at V13 were positively associated with gait speeds at V15 (β=0.022, 95%CI: 0.000,0.044).

**Conclusions:**

This SWAN sample exhibited a bidirectional relationship between gait speed and cognitive performance across time. Weighted estimates exceeded unweighted estimates, suggesting that models not accounting for selection bias underestimate associations. Future research will examine the longitudinal associations between specific domains of cognition and other measures of physical functioning among SWAN women.

